# Cannabidiol: Bridge between Antioxidant Effect, Cellular Protection, and Cognitive and Physical Performance

**DOI:** 10.3390/antiox12020485

**Published:** 2023-02-14

**Authors:** George Jîtcă, Bianca E. Ősz, Camil E. Vari, Carmen-Maria Rusz, Amelia Tero-Vescan, Amalia Pușcaș

**Affiliations:** 1 Department of Pharmacology and Clinical Pharmacy, Faculty of Pharmacy, George Emil Palade University of Medicine, Pharmacy, Science and Technology of Târgu Mureș, 540139 Târgu Mureș, Romania; 2 Doctoral School of Medicine and Pharmacy, I.O.S.U.D, George Emil Palade University of Medicine, Pharmacy, Science and Technology of Târgu Mureș, 540139 Târgu Mureș, Romania; 3 Department of Biochemistry, Faculty of Pharmacy, George Emil Palade University of Medicine, Pharmacy, Science and Technology of Târgu Mureș, 540139 Târgu Mureș, Romania

**Keywords:** cannabidiol, antioxidant, oxidative stress, nutraceuticals, physical activity

## Abstract

The literature provides scientific evidence for the beneficial effects of cannabidiol (CBD), and these effects extend beyond epilepsy treatment (e.g., Lennox–Gastaut and Dravet syndromes), notably the influence on oxidative status, neurodegeneration, cellular protection, cognitive function, and physical performance. However, products containing CBD are not allowed to be marketed everywhere in the world, which may ultimately have a negative effect on health as a result of the uncontrolled CBD market. After the isolation of CBD follows the discovery of CB1 and CB2 receptors and the main enzymatic components (diacylglycerol lipase (DAG lipase), monoacyl glycerol lipase (MAGL), fatty acid amino hydrolase (FAAH)). At the same time, the antioxidant potential of CBD is due not only to the molecular structure but also to the fact that this compound increases the expression of the main endogenous antioxidant systems, superoxide dismutase (SOD), and glutathione peroxidase (GPx), through the nuclear complex erythroid 2-related factor (Nrf2)/Keep1. Regarding the role in the control of inflammation, this function is exercised by inhibiting (nuclear factor kappa B) NF-κB, and also the genes that encode the expression of molecules with a pro-inflammatory role (cytokines and metalloproteinases). The other effects of CBD on cognitive function and physical performance should not be excluded. In conclusion, the CBD market needs to be regulated more thoroughly, given the previously listed properties, with the mention that the safety profile is a very good one.

## 1. Introduction

In light of the increased interest in natural products and the emergence of a self-medication attitude among the population, more awareness should be raised around a relatively new concept related to the natural compounds of various origins (fruits, vegetables, algae) included in food or in various pharmaceutical forms, with the aim of providing health benefits (treatment or prevention). Namely, these are substances considered nutraceuticals [1]. Consequently, the nutraceuticals market follows a similar upward trend [2,3]. There is a degree of controversy over nutraceuticals, as these are neither nutritious nor pharmacologically active, yet are assumed to be safe. In addition, the regulation of these compounds differs from country to country, and when it comes to other substances that may seem illegal at first glance (e.g., cannabidiol) their marketing may even be banned because these are considered substances of abuse. Since the regulation of the use of nutraceuticals and/or cannabidiol is not the subject of this article, for a review see [4].

The use of the *cannabis* plant for various purposes represents an increasingly frequent topic, and at the same time increasingly controversial, given its composition and rising rate of consumption. In various oriental cultures (e.g., India, China) the use of this plant is mentioned since ancient times for medicinal purposes, to treat various conditions such as pain, constipation, epilepsy, or bacterial conditions such as malaria or tuberculosis [5]. Its use is also mentioned in other conditions, this time in the psychiatric area, such as depression, and anxiety [6,7], and also as a tranquilizer and hypnotic [8,9]. Despite these considerations, the use of cannabis was considered an illegal practice, given the fact that compounds specific to the conditions in question were developed.

The *cannabis* plant has a complex composition that also includes terpenes, amides, carbohydrates, fatty acids, phytosterols, and specific compounds considered cannabinoids (cannabigerol (CBG), cannabichromene (CBC), cannabinol (CBN)) [10,11,12].

There are three species of cannabis and cultivars differ widely in their Δ^9^-THC and CBD levels as described below:

- Type I, with high content of Δ^9^-THC;
- Type II, containing different ratios of both CBD and Δ^9^-THC (predominantly CBD);
- Type III, with high content of CBD, and low content in Δ^9^-THC;

In the 1980s, following some in vitro experiments, the cannabinoid receptor type 1 (CB1) was discovered, and then, in 1990, it was also identified in the human brain. Additionally, in this period, the existence of another receptor subtype was demonstrated, namely the CB2 receptor. Following these discoveries, endogenous compounds that can stimulate these receptors were also isolated, such as anandamide (N-arachidonylethanolamine AEA, Sanskrit name, which means *internal bliss*, isolated from pig brain) and 2-arachidonylglycerol (2-AG, isolated from rat brain and canine gut) [13]. Therefore, the discovery of the two components (receptors and endocannabinoids) as well as other enzyme components (diacylglycerol lipase (DAG lipase), alpha/beta-hydrolase domain containing 6/2-arachidonoylglycerol hydrolase (ABHD6), N-arachidonoyl phosphatidyl ethanolamine phospholipase D (NAPE-PLD), monoacyl glycerol lipase (MAGL), fatty acid amino hydrolase (FAAH)) involved in synthesis or metabolism, led to the discovery of the endocannabinoid system [14,15,16,17,18].

Thus, the endocannabinoid system has an important role in the development of the central nervous system (CNS) and the neural circuits and also appears to be interconnected with other signaling pathways involved in the regulation of multiple neural functions, cognition, control of motor function, modulation of pain, and/or eating behavior [19,20,21]. Because this aspect is beyond the scope of this review, the presentation of cannabinoid receptors, CB1 and CB2 will be briefly presented in this paragraph. These two receptors are coupled with Gi/o proteins (GPCRs) with an inhibitory role and have the ability to reduce the activity of adenylate cyclase and some voltage-dependent calcium channels, but also to increase the enzymatic activity of mitogen-activated protein kinases (MAPKs) and inwardly rectifying potassium channels (GIRKs) [22,23].

As a localization, CB1 receptors are found in the liver, adipose tissue, skin, and predominantly in the CNS (cortex, hippocampus, caudate-putamen, substantia nigra pars reticulata, globus pallidus, cerebellum, spinal cord), especially in GABAergic interneurons, and glutamatergic, cholinergic, glycinergic, adrenergic, opioid, cholecystokinin, and finally serotoninergic, in the synaptic endings [20,24]. These receptors are also found on astrocytes, oligodendrocytes, and microglia, but their exact role is not defined [21]. CB2 receptors are located in the cells of the immune system [25], in microglia [26], in the vasculature [27], in pancreatic beta cells [28], at the central level, in case of the existence of a pathological condition (neuroinflammation) [29], and are also involved in the processes of atherosclerosis and bone remodeling [30]. It is important to mention that the endocannabinoid system plays a key role as an inhibitory/stimulating neuromodulator, yet its mechanism is not completely defined. Targeted areas are located both peripherally and centrally, especially in brain regions involved in affective behavior. However, in psychiatric conditions, dysregulation in endocannabinoid-mediated cell signaling could be incriminated. A hyperfunction of the endocannabinoid system associated with a massive expression of CB1 receptors was observed in the case of suicidal patients diagnosed with depression. In contrast, patients diagnosed with major depression and anxiety had lower receptor density. Concomitantly, increased AEA, peripheral brain-derived neurotrophic factor (BDNF) in physically active female patients is observed, which suggests the important role of physical exercise in neuroplasticity (and depression), directly linked to the endocannabinoid system, for a review see [31,32,33,34,35].

Regarding the mechanism of action of the endocannabinoid system, it is based on retrograde signaling. Thus, postsynaptic activity causes the production of endocannabinoids that travel to the presynaptic level and stimulate CB1 receptors with the consecutive inhibition of neurotransmitter release. There are also data in the literature characterizing non-retrograde signaling with modulation of neuronal function via transient receptor potential vanilloid receptor type 1 (TRPV1) and CB1 [36].

Based on the previously mentioned considerations, the purpose of this review is to create an image of the mechanisms by which nonpsychoactive compounds, such as CBD, regulate oxidative status, cognitive function, and physical performance are considered nutraceutical molecules.

## 2. Cannabidiol: Pharmacological Targets

In 1970 in Israel, Raphel Mechoulam discovered other compounds, called cannabinoids, that interfere with the activity of Δ^9^-THC, especially CBD, which is found in considerable amounts in *Cannabis sativa*. This molecule consists of a core of phenolic terpenes, consisting of 21 carbon atoms. CBD is one of the cannabinoids holding an important therapeutic potential among the multitude of compounds with null psychoactive properties. This derives from preclinical studies, which attest to the presence of antioxidant, neuro- and cardioprotective, anxiolytic, and anti-inflammatory characteristics [37,38,39,40]. Clinical trials involving CBD and monitoring its effect are scarce. At the same time, many of the preparations containing this compound are not approved by the authorities; the only preparation accepted by the Food and Drug Administration (FDA), which is found on the market, is Epidiolex^®^ (GW Pharmaceuticals, Cambridge, UK). Despite the fact that cannabinoid receptors are widely distributed in the body, CBD acts as a negative allosteric modulator at the CB1 level [41,42,43,44] and as an inverse agonist on CB2 receptors, through which the anti-inflammatory, reduction in self-administration of cocaine, and antiepileptic actions can be explained [41,45,46,47]. Another proposed mechanism of action is represented by the inhibition of FAAH, with the consequent increase in AEA [48].

Even if the action on cannabinoid receptors is limited, other receptors have been identified through which the pharmacodynamic properties of CBD can be explained. Thus, the affinity and agonistic activity of the TRPV1 receptor was described [48]. The literature also shows that CBD activates other vanilloid receptor subtypes (TRPV1, TRPV2, TRPV3, and TRPV4), transient receptor potential ankyrin 1 (TRPA1) [49,50,51], but the neuroprotective activity is limited to the TRPV1 receptor [52]. The CBD-induced effects which are not directly related to CB1 and/or CB2 receptors, but with transient receptor potential channels are an important topic to be discussed since it has been demonstrated that the CBD activation of receptors such as TRPV1 and TRPV2 produces several effects. Therefore, animal studies show anxiolytic, analgesic, anti-inflammatory, and anticonvulsant effects correlated with TRPV1 receptor activation. Studies carried out on different cell cultures demonstrated a reduction in inflammatory processes, consequent to diminishing levels of IL-6, IL-8, and TNF-α. CBD is the phytocannabinoid that seems to have the highest affinity to the TRPV2 receptor, and consequently the highest potency, having a pro-apoptotic effect. In contrast, the link between CBD and the TRPV3 receptor is not sufficiently studied, with little data available in the literature. Besides TRP channels, CBD acts as an allosteric modulator of GABA_A_ receptors and also presents an inhibitory action over Na^+^ channels, for a review see [53].

In addition, studies on rodents state that the stimulation of 5-hydroxytryptamine subtype 1A (5-HT_1A_) receptors is responsible for alleviating anxiolytic behavior, fear-associated freezing behavior, reducing the autonomic stress response [54], and also for inhibiting 5-HT_3_ receptors. Other therapeutic targets include protein structures involved in Ca^2+^ homeostasis, G Protein-Coupled Receptors 55 (GPR55), GPR18, and GPR119 receptors (antagonistic action), and GPR3, GPR6, and GPR12 (inverse agonistic action), glycinergic receptors *α*1 and *α*1*β*, peroxisome proliferator-activated receptor gamma (PPAR*γ*), adenosine receptors A_1_ and A_2_, lipoxygenase and cyclooxygenase type 2 (COX2) [55,56].

Regarding the therapeutic purpose of CBD, its use extends to multiple psychiatric and neurological pathologies. Perhaps the most frequently mentioned therapeutic purpose of CBD is anxiety, its anxiolytic effect being demonstrated in various clinical studies [57,58,59,60,61]. As presented throughout this review, the endocannabinoid system acts as an inhibitor through retrograde signaling at synaptic levels of GABAergic and glutamatergic systems and thus can have both anxiolytic and anxiogenic effects [62,63]. Nevertheless, the influence on other neurons should not be excluded. Such neurons are the ones that express the D_1_ dopaminergic receptors, though the exact connection is not completely elucidated. The D_1_ receptors are located in CNS regions (hippocampus, amygdala, nucleus accumbens) that are responsible for memorization processes and aversive behavior, and the alteration of the endocannabinoid system can affect the abovementioned processes and behaviors [64,65].

In this context of psychiatric diseases, the use of CBD for schizophrenia is also described in the literature [66,67,68]. Concrete examples of CBD involvement in schizophrenia are found in studies that indicate that CBD regulates dopaminergic activity in the mesolimbic system, alleviating the behavioral effects, though the mechanism through which D_2_ receptors expression is regulated is not clearly defined [69,70,71]. The possible partial agonist activity of CBD towards D_2_ receptors must be mentioned [72]. Unlike classical compounds used for the treatment of schizophrenia, CBD presents the advantages of improving cognitive impairments and a better safety profile. Moreover, a study conducted by Stark et al. on an animal model of schizophrenia (methylazoxymethanol acetate), revealed that CBD, administered in doses that prevent the disease onset can normalize the upregulation of D_3_ receptors in different regions of the brain by regulating the expression of this receptor [73,74].

Regarding neurological disorders, most pieces of evidence support the use of CBD in epilepsy, which is why the only existing product in CBD-based therapy, Epidiolex^®^, was approved [75,76,77]. Beyond this condition, CBD’s effects on Parkinson’s and Huntington’s disease have been intensively researched. The obtained data support the improvement of Parkinsonian symptoms, but more research in this field is needed [78,79]. In addition, preclinical studies in vitro and on animal models (rodents) support and demonstrate a beneficial effect of CBD in Alzheimer’s disease (AD), with the reduction in neuroinflammation and at the same time the promotion of neurogenesis. The proposed mechanisms are related to the inhibition of the production of pro-inflammatory cytokines (IL-1*β*, IL-6, TNF-*α*) by blocking Nuclear Factor-Κappa B (NF-κB), reducing the level of reactive oxygen species (ROS), and reducing *β*-amyloid peptide synthesis and apoptosis, as a result of PPAR*γ* activation [80,81,82].

## 3. Link between Cannabidiol and Oxidative Stress

This beneficial effect of CBD on pathological conditions (mainly neurodegenerative diseases) has been and is increasingly being exploited, which is why it is interesting to see the mechanisms by which this compound modulates the oxidative status, considering that these conditions are characterized among others by the presence of oxidative stress. The antioxidant potential is a result of the molecular structure of CBD, which converts reactive species into compounds with weaker or inert reactivity. The aromatic nucleus (which confers the molecule electrophilic character) and the hydroxyl group on the phenolic nucleus give the antioxidant properties, as demonstrated by Hampson et al., by the fact that CBD behaves similarly to known antioxidants (vitamin E, butylated hydroxytoluene, and BHT) [83,84,85].

As we can infer from the above paragraph, the idea is suggested that CBD exerts its antioxidant activity both through a direct mechanism (which is directly related to the molecular structure) and indirectly through influencing some molecular mechanisms involved in the regulation of redox homeostasis. Thus, CBD decreases the production of ROS primarily through the property of chelating transition metal ions that enter the Fenton reaction, from which free radicals result [86,87]. Associated with this mechanism, CBD increases the gene expression of the main endogenous antioxidant systems, superoxide dismutase (SOD) and glutathione peroxidase (GPx), via the nuclear erythroid 2-related factor (Nrf2)/Keap1 complex [88,89,90,91,92]. Additionally, CBD prevents the depletion of Zn and Se, which are known to have an important involvement in the enzymatic activity of SOD and GPx, respectively [93], and this antioxidant activity is assumed to be greater compared to vitamin E and vitamin C [94]. One of the most common reactions involving oxidative stress is the peroxidation of lipids, resulting in malondialdehyde (MDA) and 4-hydroxynonenal (4-HNE), compounds that provide electrophilic character and can bind to DNA, lipids, proteins and may decrease the ratio of reduced glutathione/oxidized glutathione (GSH/GSSG) [95,96]. In addition, the presence of these molecules, along with the changes they induce in other structures (formation of sulfoxides and disulfides from cysteine, kynurenine from tryptophane, tyrosine nitration), alters cellular signaling [97,98,99]. The 3-hydroxykynurenine/kynurenic acid ratio is a marker of neurotoxicity, also characterizing the oxidative status [100]. Many studies that attest to the antioxidant effect of CBD manifested in the brain are reported in the literature. This effect is demonstrated by the low level of MDA resulting from hypoxia and reperfusion conditions, respectively, and the decreased level of degraded proteins in the case of the administration of compounds with oxidant potential [101,102,103]. On the same note, in an AD model, CBD reduced the level of polyunsaturated fatty acids (PUFA) cyclization products [104].

**Figure 1:**
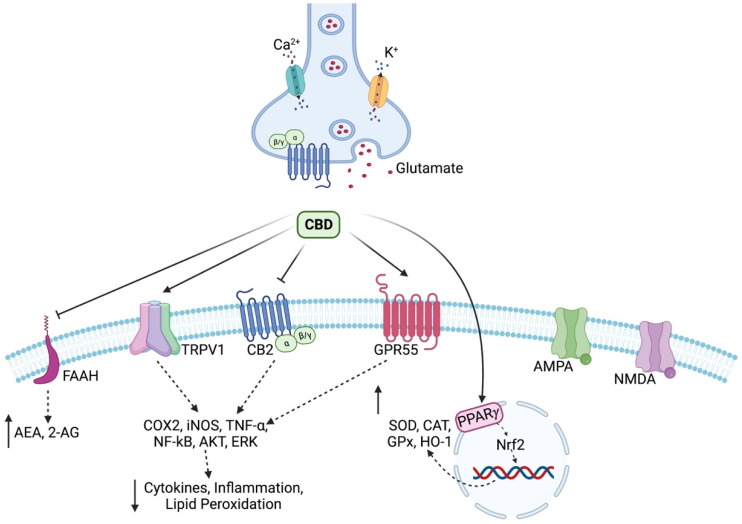
Cannabidiol has the property of inhibiting the fatty acid amide hydrolase (FAAH) enzyme with a consequent increase in the availability of anandamide (AEA) and 2-arachidonoylglycerol (2-AG), which are substances of the endocannabinoid system considered to be reverse mediators of depolarization-induced inhibition. Thus, it prevents the release of glutamate, which no longer acts on AMPA and NMDA receptors. At the same time, CBD, through TRPV1, GPR55, and CB2 receptors, prevents the generation of cytokines with a pro-inflammatory role, and in addition, through the PPAR*γ* receptor, it increases the expression of endogenous antioxidant systems via the Nrf2 pathway. Created with BioRender.com.

The cannabinoid receptor-mediated antioxidant mechanism of CBD has received limited attention due to the fact that the stimulation of CB1 receptors increases the production of ROS and TNF-*α* [105]. On CB2 receptors, CBD exerts a weak agonist action, but data suggest an inverse agonist action [106,107]. The thing that drew attention to the latter receptor is the result of its activation, exerting opposite effects of the stimulation of CB1; namely, it produces a decrease in ROS and TNF-*α* [105]. The involvement in maintaining redox homeostasis is also carried out through other receptor pathways. For example, a link between CBD binding to TRPV1 and oxidative stress is suggested, as ROS can influence the activity of this receptor by oxidizing thiol groups [108,109]. Through TRP, CBD regulates Ca^2+^ homeostasis, important in regulating the inflammatory response, via the nuclear factor of the activated T cells (NFAT) pathway [110,111]. Of major importance is that CBD stimulates PPAR*γ*, the receptor through which the transcription of pro-inflammatory proteins (COX2) is inhibited. Additionally, it inhibits other factors involved in inflammatory response signaling (NF-κB). In addition, PPAR*γ* cooperates with Nrf2 and demonstrates its cytoprotective properties by binding to the specific region of the genes encoding the antioxidant proteins, catalase (CAT), Mn-SOD, and heme-oxygenase-1 (HO-1). Last but not least, it is important to state that PPAR*γ* expression is controlled by Nrf2, by binding the latter to the antioxidant response element (ARE) sequence [112,113,114,115]. CBD also demonstrates its antioxidant properties by inhibiting the degradation of AEA and 2-AG, which are otherwise known to stimulate PPAR*γ* [116]. An indirect way for CBD to reduce the generation of ROS is represented by the GPR55 receptor, towards which it has an antagonistic behavior and can thus modulate the level of Ca^2+^, depending on the excitability of the neuronal cell, which is important in the case of pathologies such as epilepsy or AD [117]. Another receptor to which CBD shows affinity is the 5-HT_1A_ membrane receptor, through which it limits the oxidative changes resulting from the lipid peroxidation reaction [118,119]. Moreover, the fact that CBD has the ability to activate adenosine A_2A_ receptors should not be excluded. Following the activation of these receptors, the degree of oxidative damage can be improved as a result of reperfusion and it reduces the level of vascular cell adhesion molecules (VCAM-1), which gives anti-inflammatory properties in the case of multiple sclerosis [120]. Figure 1 shows the general mechanisms underlying the antioxidant properties.

Apart from the mechanisms proposed and described above, other data attesting to the antioxidant effect of CBD are presented in the literature. Thus, the induction of HO-1 by CBD [121,122,123], and the regulation of the GSH/GSSG ratio, by increasing the level of GSH, as well as SOD and GPx, is demonstrated in various experimental models, affecting both the CNS and other organs [93,101]. On the same note, it is stated that CBD decreases the expression of several isoforms of reduced nicotinamide adenine dinucleotide phosphate oxidase form (NOX) in various experimental models testing antioxidant properties [124,125,126]. Additionally, CBD reduces the general stress of reactive nitrogen species (RNS) and decreases the expression of Fas ligand (after binding to the specific receptor initiates apoptosis) and caspase-3 [93]. Last but not least, the reduction in ROS through CBD also protects the other non-enzymatic antioxidant mechanisms, represented by vitamins, with the ultimate goal of improving the oxidative status of the entire organism. One thing that should not be excluded is the oxidative status that the cell possesses, knowing that this is not always a negative factor [99], as suggested in a study led by Massi et al., who noted that CBD exerts its anti-proliferative properties depending on the level of ROS; thus, in human glioma cells, they show pro-oxidant properties, whereas, in non-transformed glial cells, they do not behave in this way [127]. These pro-oxidant properties are also dose-dependent and, paradoxically, may enhance the activity of antioxidant systems, which is important, especially in neurons [128].

## 4. Cannabidiol Involvement in Neurodegeneration and Cellular Protection

The link between neurodegeneration and oxidative stress (regardless of its origin, mitochondria, arachidonic acid metabolism, nitric oxide synthase, xanthine oxidase (XO), NOX, etc.) is a long-debated topic and the involvement of reactive species in these pathologies is generally accepted. A question that derives from the previous topic is whether ROS are at the origin of neurodegeneration, the generated reactive species are responsible for disease progression, or both. Moreover, glutamate-induced neurotoxicity is not only limited to the presence of Ca^2+^ and the activation of caspases, but also through the inhibition of the cystine/glutamate antiport (x_c_^−^), with the consequent decrease in GSH and the accumulation of ROS [129]. CBD reduces the degree of ROS-mediated neurotoxic damage in the cortical region, regardless of its origin, whether it is generated by N-methyl-D-aspartate (NMDA) receptors, α-amino-3-hydroxy-5-methyl-4-isoxazolepropionic acid (AMPA) or kainate, which suggests either an inhibitory action on these receptors or an action on downstream proteins [112]. At the same time, previous studies have suggested and even stated that CBD has an antioxidant capacity similar to BHT, and in addition, it does not promote protumor effects, thus making it one of the most promising agents in this regard [83]. For example, in Parkinson’s disease (PD), the region affected by ROS is considered to be the substantia nigra pars compacta (SNpc), where the highest density of dopaminergic neurons is found, where the basal level of ROS is higher than in other areas of the brain, as a result of the intense metabolism of dopamine. Thus, these neurons are susceptible to the presence of oxidative stress, the decrease in their number causing the appearance of motor, but also cognitive, memory, and learning symptoms [99]. Thus, the addition of CBD in therapy increases the number of unaffected dopaminergic neurons, also suggesting the importance of the HO-1 system in neuroprotection [123]. At the same time, the continuous administration of CBD in preclinical studies led to the observation of decreased levels of MDA at the cortical and striatal level, as well as increased activity of antioxidant mechanisms in other areas (cortex and striatum) [130]. Similar effects could also be observed in various preclinical models of PD [131,132].

The presence of neuroinflammation within controlled limits is imperative for the restoration of neuronal tissue, but such a particular state continues to be associated with the development of neurodegenerative diseases [133]. Without a well-known mechanism, the density of CB2 receptors in microglia increases under oxidative stress conditions, as seen in multiple sclerosis, AD, and PD. The activation of these receptors causes the inhibition of microglia activity, hence the reduction in neuronal toxicity mediated by ROS and RNS [134,135], by decreasing glial fibrillary acidic protein expression (GFAP) [136]. At the same time, CB2 activation improves blood–brain barrier (BBB) function and reduces inducible nitric oxide synthase (iNOS) expression as a result of extracellular signal-regulated kinase 1/2 inhibition (ERK 1/2) [137]. It is suggested that CBD exerts its neuroprotective and antioxidant properties in a unique manner that is independent of CB2, TRPV1, or PPAR*γ* receptors. In experimental studies aimed at testing the effect of CBD in AD, it reduced iNOS activity, decreased tau protein hyperphosphorylation, MDA levels, and caspase 3 activity [138]. In the case of Huntington’s disease, 3-nitropropionic acid (3-NP) is used to generate a model as close as possible to the pathology. The administration of CBD led to the rescue of neurons, independent of CB2 receptors, the most likely reason being the antioxidant effect [139].

Other mechanisms involved in the control of inflammation are the inhibition of NF-κB and also of the genes that encode the expression of molecules with a pro-inflammatory role (iNOS, COX2, cytokines, and metalloproteinases) [140]. Additionally, the inhibitory control of NF-κB can be achieved by reducing the phosphorylation of kinases involved in the transcription of this factor such as p38 mitogen-activated protein kinase (p38 MAPK) [137]. Additionally, in the same category of mechanisms with neuroprotective potential are included 5-HT_1A_ receptors, adenosine reuptake inhibition, and the WNT/*β*-catenin signaling pathway, which has an important role in *β*-amyloid (A*β*)-induced glycogen synthase kinase-3 beta activation (GSK-3*β*) and tau hyperphosphorylation [141,142].

Several studies have focused on investigating how CBD works in neurodegenerative diseases. Thus, in animal models (rats) of AD, CBD treatment prevented cognitive impairments and reduced the risk of progression via PPAR*γ* and SOD. It is also not excluded that AEA, whose level increases in the presence of CBD, and reduces the formation of *β*-amyloid, as suggested in cell culture studies, also has an important role. As previously stated, other studies also support the inhibition of the GSK-3*β* protein complex through TRPV1-mediated phosphoinositide-3-kinase/protein kinase B (PI3K/Akt) activation and protection of neuronal plasticity by reducing ROS and inflammation [143,144,145,146].

Literature reports suggest that the accumulation of A*β* destabilizes the redox balance, generates massive amounts of ROS (promotes lipid peroxidation reactions, protein, and DNA oxidation) [147], and stimulates pro-inflammatory reactions, which ultimately result in disease progression [148]. On the same note, NF-κB, a factor sensitive to changes in oxidative homeostasis, which is activated by the family of stress-activated protein kinases (SAPKs), which includes p38 MAPK, regulates the transcription of pro-inflammatory factors and the immune response, but also induces iNOS in neurons affected by the presence of A*β*, both in cell cultures and in post-mortem studies on the brains of AD patients [149,150,151]. Thus, it is demonstrated that CBD exerts an inhibitory effect on both NF-κB and p38 MAPK, ultimately determining the reduction in iNOS expression with the limitation of the disruptive effects of oxidative stress, and at the same time being responsible for the protection of PC12 cells from the negative effect of A*β* [137]. The idea is strengthened by the fact that CBD protects PC12 neurons in an antioxidant manner and also reduces the hyperphosphorylation of tau proteins through the WNT/*β*-catenin pathway [112], diminishing the inflammatory markers generated by A*β*, such as GFAP and IL-1*β*, these effects being the sum of complex and dynamic processes [152,153,154,155]. Another cause of the vicious circle created by ROS and the progression of A*β* plaque aggregation (which generates in vitro, H_2_O_2_, and $O_{2}^{-}$), is the presence of increased concentrations of transition metal ions (Cu^2+^, Fe^3+^) in the brain of patients (postmortem) diagnosed with AD [156,157,158]. In this sense, the chelating properties of CBD, along with the antioxidant ones, can constitute an alternative treatment [159].

In the case of cell cultures (PC12 cell line), treated with MPP^+^, the inclusion of CBD under these conditions resulted in increased viability of these cells and also the expression of the axonal protein, Growth Associated Protein 43 (GAP-43). CBD also stimulates neurite formation but, in a manner, is closely related to the tropomyosin kinase A receptor (TrkA) and not to the nerve growth factor (NGF) [157]. Depending on the time of administration, i.e., the time elapsed since the injury occurred, CBD has the ability to restore the dopamine level, as demonstrated in the case of the injection of 6-hydroxydopamine (6-OHDA), and is proposed as a mechanism, the activation of the antioxidant systems and CB2 receptors, respectively, TRPV1 expressed at nigrostriatal level, as well as COX2 inhibition [132].

Preclinical models based on 3-NP induce striatal toxicity, but apparently, CBD can increase gamma-aminobutyric acid (GABA) levels and protect GABAergic neuron projections in the SN, increase BDNF, reduce ROS, and also reduce cellular signaling via the PI3K/Akt pathway, independent of CB2 or TRPV1 receptors, whereas the reduction in iNOS is mediated by CB2 [102,139,160,161,162]. It is very likely that in this case too, the beneficial effects of CBD are mediated through the Nrf2/ARE pathway, as it is known that in the case of 3-NP intoxication, the expression of Nrf2 is increased [163,164,165].

An important aspect to specify is that oxidative stress is not only found in neurodegenerative conditions but also in particular psychiatric conditions. Thus, regarding ROS, it doesn’t need to constitute the origin of these conditions, but rather the aggravation of symptoms and brain functions. In this sense, mental states characterized by stress induce ROS by altering the balance of excitatory neurotransmitters (adrenaline, glutamate), serotonin, and GABA, which also modulate the immune response and maintain the body’s homeostasis and the functionality of the hypothalamic-pituitary-adrenal axis (HPA) [166]. For this purpose, in post-traumatic stress syndrome (PTSD), CBD finds applicability by blocking FAAH and increasing available AEA [167,168,169]; in depression, CBD alleviates symptoms by stimulating serotoninergic neurotransmission, mediated by 5-HT_1A_, and by activating the BDNF-TrkB complex [170,171,172], whereas in anxiety and fear, these beneficial effects are mediated by the same 5-HT_1A_ receptor, but also by GABA_A_ receptors, respectively, by inhibiting the enzymatic activity of iNOS and FAAH [173].

These positive effects exerted by CBD (antioxidant, neuroprotective) have also been investigated outside the context of neurodegenerative diseases. Thus, in the case of toxicity mediated by alcohol consumption (in the rat binge alcohol model), CBD limited the pathological changes in the hippocampal region, the proposed mechanism is based on antagonizing the effect of glutamate, beyond the stimulation of NMDA receptors (by glutamate), being demonstrated neuronal decline by alcohol, through the influence on mitochondria, resulting in ROS [174]. Additionally, in a cardiotoxicity model using doxorubicin, CBD limited this effect by enhancing mitochondrial complex I activity and also GPx activity [175]. The same antioxidant behavior of CBD could also be observed in a cisplatin-induced nephrotoxicity model [124].

Another area of interest in the use of CBD is the effect of this compound on the skin. Thus, it is known that ultraviolet radiations (UV), UVA, and UVB produce a redox imbalance, which results in the degradation of the normal structure of the skin with possible consequences such as photoaging and photocarcinogenesis [176,177]. Apparently, at this level as well (keratinocytes), CBD can induce Nrf2 and decrease ROS. In addition, an increase in the level of thioredoxin and the activity of thioredoxin reductase is observed, a system through which the activity of apoptosis-regulating kinase (ASK-1) is inhibited, thus protecting cells from apoptosis induced by ROS and irradiation [178,179,180,181]. Moreover, CBD reduces the signaling of apoptotic pathways by restoring Ca^2+^ homeostasis at the mitochondrial level [166]. Under physiological conditions, CBD activates A_2_ receptors with a reduction in NF-κB activity and a consequent decrease in TNF-*α* [182,183]. Another proposed mechanism is that through which CBD stimulates the formation of an anti-inflammatory prostaglandin, 15d-PGJ2, which regulates COX activity and also the redox balance by facilitating the dissociation of Nrf2 from Keap1, an effect that promotes HO-1 transcription and finally the rescue of irradiated keratinocytes [184,185,186,187]. Related to Nrf2, it seems that continuous and prolonged activation of this factor increases the risk of malignancies and creates an environment favorable to the development, proliferation, and creation of resistance to chemotherapy and radiotherapy through antioxidant mechanisms [188,189] At the same time, it is suggested that CBD reduces the transcription of Nrf2, manifesting in this way the protection of cells [190].

**Figure 2:**
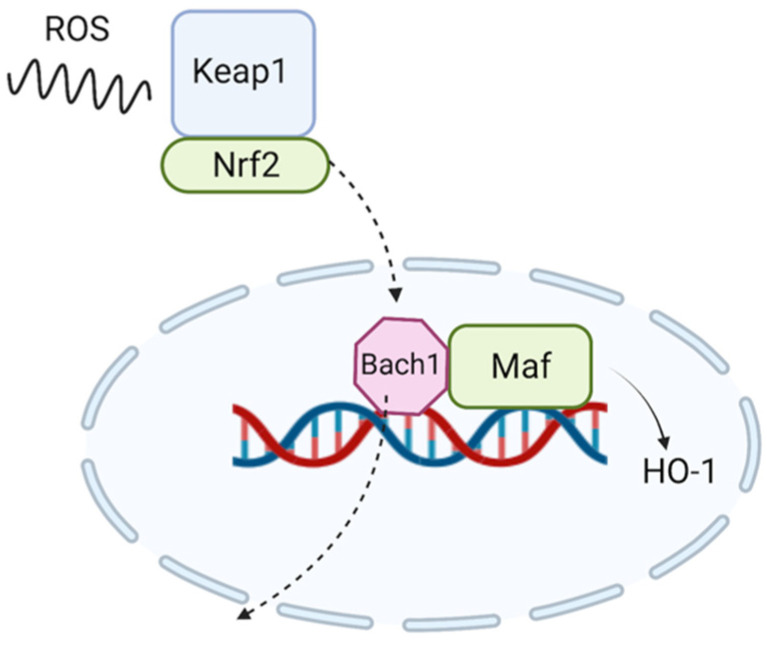
Under the action of reactive oxygen species (ROS), the Keap1-Nrf2 complex cleaves. Once cleaved, Nrf2 enters the nucleus at the DNA level, where it displaces Bach1 from Maf, in order to synthesize HO-1. Created with BioRender.com.

In addition to influencing Nrf2 and HO-1, various studies state that CBD has an inhibitory effect on BTB Domain and CNC Homolog 1 (BACH1), a transcription factor involved in ROS generation [90,121,188,191,192,193]. This BACH1 can be considered as a functional antagonist of Nrf2, in the absence of oxidative stress, by binding to Maf recognition elements (MAREs), regions indispensable for the coding of genes with an antioxidant role, following that the same Nrf2 will restore the levels of BACH1, decreased due to oxidative stress [194] (see Figure 2). Other studies claim that, in fact, in keratinocytes, CBD is a weak activator of Nrf2 and rather a strong inhibitor of BACH1 [193].

## 5. Cannabidiol and Cognitive Performance Related to Oxidative Stress

In the absence of a condition affecting cognitive function (attention, memory, ability to use memories), CBD does not show an improvement in this function. However, in the case of pathological situations that induce cognitive impairments (AD, neuroinflammatory conditions, ischemic states, epilepsy, schizophrenia, hepatic encephalopathy), studies show an improvement in these events [195,196,197,198,199]. In addition, in studies on human subjects, cannabis users with increased concentration in CBD, no memory impairments were reported [200]. Most often, glutamate toxicity is described in the inability to form and use memories, which triggers irreversible processes resulting in lipid peroxidation resulting in ROS production, vascular microlesions, and last but not least, cell death [201]. According to this hypothesis, it is suggested that CBD administration alleviates glutamate-induced toxicity by reducing available Ca^2+^. In addition, alongside this effect, others such as the antioxidant and anti-inflammatory effects can be counted, and it is also considered that the combination of THC and CBD is more effective than CBD alone [202,203,204], although there are studies that state that CBD protects memory function of the negative effects of THC [55,205] being observed that in the case of people who consume cannabis with a high content of THC and low content of CBD, they have a smaller hippocampal region, associated with low cognitive performance [206]. In a study conducted by Friedman et al., it is considered that in the case of most brain traumas, they are moderate, and cognitive impairment can be improved by applying CBD. Regarding the size of the lesions, CBD has the ability to limit this and also improve vestibulomotor function and learning as well as memory. On the other hand, what cannot be stated with certainty is whether the beneficial effects are the result of the anti-inflammatory or antioxidant effect [207].

Another proposed mechanism for improving cognitive capacities is based on antagonizing the pro-oxidant effect of transition metal ions through their chelation, a topic discussed in the previous section. In the case of the presence of pathologies affecting the brain, changes in memory function are most often observed. A study led by Fagherazzi et al. suggests that CBD acts mainly in the memory consolidation phase and not in the acquisition and retrieval phase [208]. Another study reported that CBD administration was beneficial in reversing cognitive deficits in sepsis, and this is due to its antioxidant properties [193]. Regarding this particular condition (sepsis), it is shown that ROS are generated leading to oxidative damage not only in the periphery but also in the CNS [209,210]. In this sense, the administration of CBD, due to its antioxidant properties, limits these lesions in the hippocampus, in the initial stages of sepsis, and prevents memory loss through an antioxidant mechanism, in animal models [211]. In another study, this time focusing on patients with schizophrenia, it is observed that it does not improve cognitive function, based on the consideration that the effect of CBD is crucial during critical periods of the disease, and not in chronic schizophrenia [212]. The data on this subject are quite contradictory, so Blaes et al. presented a beneficial effect of using cannabis in inhaled form (smoke), and the proposed mechanism is based on prefrontal cortex pyramidal neurons. Thus, in these neurons, GABAergic neurotransmission plays an important role [213]. Additionally, there are CB1 receptors in this area, whose primary role is to inhibit the release of GABA and promote working memory, thus preventing the suppression of pyramidal neurons by GABA [214]. This beneficial effect observed in the case of THC may be due to differences in pharmacokinetics, given the fact that by inhaling the smoke, a smaller amount of the substance is absorbed [215]. Another paper in the literature on hepatic encephalopathy shows that motor and cognitive functions are improved through the 5-HT_1A_ and A_2A_ receptor pathways [160].

An immunological mechanism with implications in AD and at the same time in cognitive performance is related to IL-33, a centrally expressed cytokine, especially in the conditions of altered BBB function. Along with this marker, the triggering receptor expressed on myeloid cells 2 (TREM2), is another important factor in the development of AD, suggesting that the latter has a neuroprotective role. In preclinical studies on mice, a considerable increase in IL-33 and TREM2 could be observed after CBD administration, with a parallel decrease in A*β* production, while also improving contextual memory [215]. Beneficial effects on memory could also be noted in an animal model of PD induced by reserpine treatment [216]. In the case of the study conducted by Razavi et al., CBD treatment during methamphetamine-induced abstinence resulted in improved spatial memory [217]. These data are evidence that CBD intervenes in cognitive processes and through antioxidant mechanisms, knowing the potential of amphetamines to generate oxidative stress [103]. Concerning drug addiction, in part to substances in the class of amphetamines, CBD seems to have the ability to attenuate the hyperactivity of dopaminergic neurotransmission in the mesolimbic region through an agonistic behavior towards dopaminergic D_2_ receptors [218].

The study conducted by García-Baos et al. demonstrates the protective effect of CBD on cognitive function (effect exerted in the hippocampal region and the prefrontal cortex) in the case of mice exposed to alcohol during the prenatal period, respectively, during the lactation period, most likely through the mediation of NF-κB, TNF-*α*, COX2, and caspase-3 expression, events also noted in other studies [219,220,221,222], but also in ischemia situations [223,224]. The same study states that CBD does not improve recognition memory, but rather spatial memory, assumptions reinforced by other studies in this field [205,219,225]. Another possible mechanism is the inhibition of AEA metabolism. Thus, the two molecules (CBD and AEA) can activate the TRPV1 receptors and cause glutamate release [226]. Table 1 states the results of studies on cognitive function after CBD administration.

## 6. Cannabidiol and Physical Performance

Regarding CBD use in athletes, the World Anti-Doping Agency (WADA) has included this compound in the list of substances prohibited during competitions [231]. Inclusion on this list is based on the hypothesis that CBD possesses the ability to enhance physical performance through various mechanisms [232,233,234]. A detail that must be considered is that there is a risk that CBD-containing products are also contaminated with THC (a compound found on the same prohibited list), which would result in a positive result in the anti-doping test [235,236].

Although multiple studies in the literature suggest improved physical performance as a result of the calming/relaxing effect of CBD [60,237,238], the present study focuses on antioxidant and muscle recovery mechanisms. Thus, it is known that high-intensity physical exercise correlates with inflammation and damage of skeletal muscle, and at the same time, with a decrease in physical performance [239]. For these reasons, athletes seek and resort to methods that shorten their recovery period [240,241,242]. These beneficial effects on muscle appear to be due to the inhibition of NF-κB and activation of the JAK/STAT (Janus Kinase/Signal Transducer and Activator of Transcription) pathway. However, a study conducted by Isenmann et al. noted that a single dose of CBD had minimal effect on creatine kinase (CK) and myoglobin levels after 72 h. It is therefore suggested that for a more pronounced effect, multiple doses are likely to be more effective [243]. In addition, anti-inflammatory properties are correlated with decreased levels of cytokines, prostaglandin E2 (PGE2), and NO, and are assumed to be mediated by 5-HT_1A_ and TRPV1 receptors [244]. In another study, this time conducted by Ianotti et al., in a model of muscular dystrophy (Duchenne muscular dystrophy), it was shown that the use of CBD limited motor dysfunction, improved muscle strength, and also reduced pro-inflammatory markers (IL-6, TNF-*α*, TGF-*β*1, iNOS) and autophagy (autophagy-regulating protease 4 and 12 (Atg4, Atg12), Unc-51 like autophagy activating kinase (ULK1)). In the same study, the authors observed that on C2C12 myoblasts, CBD exerted its pro-differentiating effect via TRPV1 [245,246]. At the same time, in a study on human subjects, to evaluate soreness and performance, it was observed that the administration of CBD (150 mg), regardless of the time of administration (24 and 48 h) did not lead to an improvement in muscle condition [247], whereas, in another study, the administration of CBD (16.67 mg given with 1 mL of medium chain triglyceride) at 24, 48, and 72 h visibly reduced the muscle pain felt [248].

These considerations are based on the idea that inflammation, exercise-related damage proliferation, and cell differentiation are closely related to ROS, which gives rise to another hypothesis, that the reduction in oxidative stress can have an important role in sports [249]. On the same note, CBD, by regulating cortisol release via CB1, CB2, and A_2_ receptors, decreases the level of immune cells and cytokines (IL-1, TNF-*α*), and in addition, favors the release of arachidonic acid with the stimulation of healing capacity, promoted by growth and anti-inflammatory signals (lipoxin A4, 15d-PGJ2) [250,251].

Despite the fact that the results of the studies carried out to date do not provide conclusive data on whether the use of CBD helps or improves physical performance, thus attracting controversy regarding sports ethics, this compound seems to be increasingly used, and for this reason, more extensive studies are needed, both clinical and preclinical, to establish as clearly as possible the mechanisms, respectively, the pharmacodynamic effects in sports (when used before, during, and/or after training). Thus, these studies should consider several aspects, including the type of muscle pain, the fatigue of the individuals, the type of physical effort/sport, as well as gender differences.

## 7. Cannabidiol and Autophagy

The autophagy process represents a cellular mechanism by which damaged organelles and non-functional protein aggregates are degraded to obtain energy or recycle them (after degradation, amino acids are used for new synthesis). This is a very precise process aimed at maintaining the body’s homeostasis, normal growth, and development, as well as regulating inflammatory processes and immunity, and protecting against viral and/or bacterial infections. There are three types of autophagy (macroautophagy, microautophagy, and chaperone-mediated autophagy), but macroautophagy is currently thought to be actually autophagy [252,253,254].

Thus, in the case of neurodegenerative diseases, it has been observed that most of the time a mitochondrial dysfunction occurs that determines the progression of the disease [255]. In an experimental model of MPP^+^-induced PD in SH-SY5Y cell cultures, CBD administration protected these cells by increasing the expression of silent mating type information regulation 2 homolog 1 (SIRT1), to inhibit NF-κB. By inducing autophagy, attenuation of Tyrosine Hydroxylase (TH) loss and *α*-synuclein accumulation could be observed [256]. At the same time, it is known that in cases where mitochondrial dysfunction occurs, the expression of Nrf2 and antioxidant mechanisms are decreased, which implies a progression of oxidative damage [257]. Furthermore, pretreatment of SH-SY5Y cells with CBD mediates oxidative stress damage through the activation of PINK-1/parkin and DJ-1 proteins [256]. For this reason, the presence of the autophagic mechanism is necessary, the neurons use the energy resulting from the affected organelles, and the synaptic remodeling is also stimulated, but these beneficial effects also extend to other pathologies [252]. At the same time, the opposite extreme is not excluded, a dysregulation of autophagy can also lead to neuronal impairments and neurodegenerative diseases [258]. In a recent study, CBD-induced autophagy was concentration-dependent and required communication between extracellular signal-regulated protein kinases 1 and 2 (ERK1/2) and Akt [259]. Another subject in which autophagy is brought to the fore is the effect on the aging process of CBD, being known that autophagy is closely related to caloric restriction and the anti-aging effect, as suggested in multiple studies [260,261,262]. In a recent study on Caenorhabditis elegans, it is shown that CBD increases autophagic activity during aging and improves the health span and morphology of neurons in the context of aging [263], knowing that the cytoarchitecture of neurons changes during this process, and at the same time there is a decline of cognitive abilities [264]. Therefore, it is suggested that CBD does not necessarily improve these functions, but at least keeps them within normal limits. Another aspect that must be considered for the proper functioning of cells is mitochondrial homeostasis and the influence that CBD has on this organelle. In this case, two situations can occur in cases of high metabolic stress, namely mitochondrial fusion and fission [265]. Thus, mitochondrial fusion protects the organelle from age-related mtDNA mutations and is mediated by a protein, Optic atrophy 1 (OPA1), whereas mitochondrial fission is mediated by Dynamin-related peptide 1 (Drp-1) [99]. The study conducted by da Silva et al. evaluating iron-induced toxicity through oxidative stress in the brain demonstrates the beneficial effect of CBD by bringing the protein expression of Drp-1, caspase 3, and synaptophysin to values similar to those observed in the control group, without altering OPA1 [266]. In this regard, other studies have also shown an improvement in memory and a reduction in synaptophysin levels in cases of iron intoxication after administration of CBD [207], whereas an animal model of hypoxic-ischemic injury demonstrated a reduction in caspase 9 levels [267], and a study on PC12 cells treated with A*β* reduced caspase 3 levels [150]. Another detail that should be highlighted is the possibility that CBD manifests its effects on mitochondrial dynamics, noting that in the hippocampus Drp-1 levels are reduced, whereas OPA1 is increased [266].

These data support the idea that CBD exerts its neuroprotective effect not only through its antioxidant potential but also through its antiapoptotic properties.

## 8. Conclusions

In this article, we have highlighted the positive effects of CBD on several functions, which are closely related to its antioxidant properties and effects.

In conclusion, the endocannabinoid system and cannabinoid derivatives, such as CBD, will be increasingly researched and will certainly show more and more therapeutic importance, given the scientific evidence obtained both in preclinical studies and clinical ones.

## Figures and Tables

**Table 1:** Experimental results of cognitive behavioral tests performed on rodents.

Animal Model	Cognitive Impairment Model	Task	Treatment (Dose)	Duration	Observations	Ref.
Sabra female mice (8 weeks)	Hepatic encephalopathy (bile duct ligation)	Eight-arm maze test	CBD i.p. (5 mg/kg)	21 days	Restored cognitive function (*p* = 0.001). CBD had no effect on sham animals.	[160]
Male and female Wistar rats (7–10 days old)	Hypoxia-ischemia (left carotid artery electrocoagulation)	Novel object recognition test (NOR)	CBD s.c. (1 mg/kg)	3 days	CBD-treated rats test performance was similar to that of sham animals (*p* < 0.05).	[196]
Adult male and female Sprague–Dawley and Wistar rats	Controlled cortical contusion injury	T-maze test Novel object recognition test (NOR)	CBD i.p. (40 mg/kg, 20 mg/kg)	15 days	CBD treatment increased the discrimination index compared to the traumatic brain injury group, being able to discriminate the novel object.	[207]
Male Wistar rats (3 weeks)	Iron neonatal treatment	Inhibitory avoidance task Novel object recognition test (NOR)	CBD i.p. (2.5, 5, 10 mg/kg)	14 days	The 10 mg/kg dose of CBD was able to reverse iron-induced memory deficits, recognition index of this group being significantly higher when compared to the iron treated group (*p* < 0.0001).	[208]
Male Wistar rats (3–4 months)	Sepsis induction-cecal ligation and perforation	Inhibitory avoidance test	CBD i.p. (2.5, 5, 10 mg/kg)	9 days	CBD in all doses reverted the memory alteration (*p* = 0.001).	[209]
Male and female Long Evans rats	-	Working memory test	Cannabis cigarettes (5.6% THC, 0% CBD, 0.4% CBN)	-	In male rats, cannabis smoke exposure did not affect choice accuracy. In female rats, however, exposure to cannabis smoke significantly enhanced choice accuracy. The effect on working memory accuracy, cannabis smoke increased the number of trials completed in females (*p* = 0.01) but not in males (*p* = 0.48) and decreased locomotor activity in both sexes (males: *p* = 0.04; females; *p* = 0.03). It should be noted that the performance-enhancing effect after exposure to cannabis smoke was evident only in female rats, whose initial performance was significantly lower than in males.	[213]
5xFAD male mice (9–12 months)	Transgenic mice	Novel object recognition test (NOR)	CBD i.p. (10 mg/kg)	2 weeks (one dose every other day)	CBD treatment improved cognitive function as measured by NOR (Discrimination Index increased to 0.5 ± 0.9 from −0.2 ± 0.8, *p* ≤ 0.04).	[215]
Male Wistar rats (3 months)	Motor and cognitive impairments induced by reserpine	Plus maze discriminative avoidance task	CBD i.p (0.5 and 5 mg/kg)	4 days	The time spent in aversive enclosed arm is lower than the time spent in the non-aversive enclosed arm for CBD 0.5 (*p* < 0.05), but not for CBD 5.	[216]
Male Wistar rats	Methamphetamine chronic exposure (10 days, twice/day)	The Y-maze (YM) test Novel object recognition test (NOR)	CBD ICV microinjection (32 and 162 nmol)	10 days	Both doses of CBD significantly improved spatial memory. The higher dose of CBD was more effective, *p* < 0.001). A high dose of CBD(160 nmol) could improve long-term memory *p* < 0.025).	[217]
Male and female offspring C57BL/6J	Alcohol exposure	The Y-maze (YM) test Novel object recognition test (NOR)	CBD i.p. (20 mg/kg)	10 days	CBD-treated group showed a significantly higher preference for the novel arm as compared to alcohol-exposed group (*p* < 0.05), revealing that CBD treatment hampers the detrimental effect on reference memory caused by alcohol. CBD does not affect the recognition memory (NOR), but CBD treatment impedes the deleterious effects of alcohol on object location memory.	[222]
Male C57BL/6J mice (2–3 months)	Bilateral common carotid artery occlusion (BCCAO)	The Y-maze (YM) test	CBD i.p. (10 mg/kg)	0.5, 3, 24, 48 h after the surgery	CBD increased in the % of the time in the novel arm of the YM when compared to BCCAO animals treated only with vehicle *p* < 0.01.	[224]
Male Sprague–Dawley rats	Schizophrenia-like cognitive deficits induced by repeated ketamine administration	Novel object recognition test (NOR)	CBD i.p. (7.5 mg/kg)	6 days	Ketamine-induced cognitive deficits were restored by CBD (*p* < 0.001).	[227]
Adult female Wistar rats	Estrogen depletion (surgery)	Inhibitory avoidance	CBD i.p. (10 mg/kg)	14 days	Ovariectomized rats treated with CBD had a higher latency to step down (*p* = 0.001).	[228]
Male Wistar rats (3 weeks)	ICV injection of streptozotocin	Novel object recognition test (NOR)	CBD i.p. (20 mg/kg)	7 days	CBD-treated group showed a better performance both on short- and long-term memory (*p* < 0.0001).	[229]
Swiss male mice (4 weeks)	MK-801 injection	Passive avoidance test	CBD i.p. (1, 5, 30 mg/kg)	Acute experiment	CBD treatment in dose of 30 mg/kg appeared to have a better performance (*p* < 0.001).	[230]
